# A global dataset of UHPC mix designs with supplementary cementitious materials and nano additives

**DOI:** 10.1016/j.dib.2025.112179

**Published:** 2025-10-16

**Authors:** Umair Jalil Malik, Damith Mohotti, Huadong Mo, Chi King Lee

**Affiliations:** a School of Engineering and Technology, University of New South Wales, Canberra, Australia; b School of System and Computing, University of New South Wales, Canberra, Australia

**Keywords:** Ultra-high performance concrete, Supplementary cementitious materials, Nano particle, Mechanical properties, Durability, Predictive modelling, Global Dataset

## Abstract

The incorporation of supplementary cementitious materials, nano additives and sustainable fillers in Ultra-High Performance Concrete (UHPC) has been recognized as a valuable solution to reduce cement usage while enhancing the mechanical and durability performance. However, replacing cement with sustainable alternatives presents a significant challenge, as optimizing UHPC mix designs involves multiple parameters and extensive hit-and-trail experimentation. However, Artificial Intelligence (AI) has gained attention as a powerful approach for predicting the performance of advanced cementitious composites, enabling more efficient mix design optimization and reducing extensive laboratory testing. Despite these advancements, most available UHPC datasets are limited in scope, focusing on specific regions, narrow ranges of properties, or select mix parameters which restricts their effectiveness for global research and comprehensive data-driven modelling. To address this gap, a comprehensive global dataset of UHPC mix designs featuring 2188 mix designs from 168 publications has been compiled across a diverse range of countries, cement types, supplementary cementitious materials (SCMs), nano particles, fillers, fibre, and curing regimes. The dataset is formatted and standardized for use in machine learning (ML) model development and inverse design strategies for developing sustainable and ecofriendly UHPC. By offering a globally diverse dataset, this work provides a valuable resource for advancing the design of sustainable UHPC mixtures.

Specifications Table

**Table:** 

Subject	Engineering & Materials science
Specific subject area	Nano Particles and Supplementary Cementitious Materials based Ultra-High Performance Concrete
Type of data	Table and Figure. Data formats: Raw and analysed
Data collection	Data were collected through a comprehensive literature review of 168 publications on ultra-high-performance concrete. Experimental data including mix proportions, curing regimes, workability, mechanical, and durability properties were extracted from tables and figures in these publications. Data from figures were digitized using Web PlotDigitizer, and all values were standardized to consistent units. The data extracted were then compiled into a structural dataset based on their mix constituents, corresponding mechanical properties and durability performance. Only studies reporting complete mix design details and corresponding performance metrics were included, ensuring data reliability and comparability across the dataset.
Data source location	We provide a reference to the raw data in the following link: https://data.mendeley.com/datasets/czb7ww5pkz/2
Data accessibility	Repository name: Mendeley Data Data identification number: 10.17632/czb7ww5pkz.2 Direct URL to data: https://data.mendeley.com/datasets/czb7ww5pkz/2
Related research article	None

## 1 Value of the Data

• The entire dataset is uniquely structured into 5 principal components: i) Mix Constituents includes detailed information on the quantities and types of materials used in each UHPC mix (Cement, a wide range of supplementary cementitious materials, nano particles, fillers, fibers, aggregates (sand type and amount), water, and superplasticizer), ii) Curing Regime (Method, Temperature, Humidity and pressure), iii) Workability (Slump and Slump Flow), iv) Mechanical Properties (Compressive Strength at multiple ages (1,3,7,14,21,28,56 and 90 days), Elastic Modulus, Tensile Strength Parameters and Flexural Strength Parameters), v) Durability Performance (Porosity, Air Content, Air Voids, Shrinkage, Freezing and Thawing cycles, Rapid Chloride Permeability, Surface Resistivity and water absorption). The UHPC dataset was compiled from 168 research articles, resulting in a total of 2188 unique mix designs. The data represent diverse regional practices and offer a valuable foundation for sustainable development of UHPC.
• Most previous UHPC datasets often only cover a limited set of mix design parameters and fail to distinguish between SCM subtypes (e.g., treating fly ash, slag as a single component), and missing essential details such as curing methods or specimen dimensions [1,2]. In addition, unlike most existing datasets are region-specific and limited in scope, restricting their applicability to broader environmental or material contexts, this dataset is compiled from studies conducted worldwide, capturing the diversity of raw materials, regional practices, and environmental conditions. This diversity increases its relevance for global applications and supports to perform comparative studies, predictive modelling and optimization across multiple variables.
• The dataset was created primarily to support predictive modeling and machine learning applications in UHPC research. Most existing predictive models for UHPC have been developed using datasets that are limited in scope, often focusing on specific regions or incorporating only a narrow range of mix design variables and properties [[3], [4], [5], [6], [7]]. This comprehensive and globally diverse dataset provides a strong foundation for both forward prediction (estimating UHPC properties from mix design) and inverse design (identifying optimal mix designs for targeted properties). It enables more reliable prediction of mechanical properties and durability and supports the optimization of sustainable UHPC mixes to meet specific performance criteria. By using this dataset for inverse design development, the models can generate mix design solutions tailored to desired compressive strength or other target properties, facilitating development of formulations that achieve balance strength, durability, and sustainability.
• The inclusion of a wide array of SCMs, nano additives, and alternative sustainable fillers provides a valuable foundation for researchers exploring sustainable and high-performance UHPC formulations. The data can be employed to investigate the effects of material substitutions, assess environmental impacts, and guide the development of eco-friendly construction materials.
• The dataset is organized in such a way that new data from future studies can be added to the dataset easily. This extensible design enables continuous expansion and supports the long-term development of a comprehensive resource for UHPC-related research and practical applications, maintaining its relevance and utility for engineering.

## 2 Background

The increasing demand for sustainable construction materials and the environmental impact of conventional cement production have driven significant research into alternative approaches for UHPC [8]. SCMs (Silica Fumes, Fly ash, ground granulated blast furnace slag (GGBFS) etc.) [9], Nano additives (Nano SiO_2_, Nano CaCO_3_ etc.) [10] and sustainable fillers (Silica Flour, Steel Slag Aggregates, Iron Tailings etc.) as partial replacements for cement and sand can lead to reduction of carbon emissions and efficient use of industrial byproducts. UHPC is recognized for its exceptional mechanical strength and durability achieved through optimized mix designs that incorporate SCMs, nano additives, fibers, and chemical admixtures [11]. Despite the development of various mixture design approaches in the literature, most UHPC formulations still require extensive experimental trials, which are time-consuming and resource-intensive [12]. Machine learning (ML) provides an efficient alternative by capturing complex nonlinear relationships between mix constituents and performance outcomes [13,14]. Compared to traditional statistical approaches, ML offers higher accuracy and flexibility, enabling both forward prediction (estimating mechanical and durability properties of UHPC) and inverse design (optimizing mixes to achieve targeted performance [[15], [16], [17]]. The effectiveness of such models is closely tied to the availability of large, diverse, and standardized datasets. A significant challenge in advancing research and predictive modeling for UHPC has been the lack of comprehensive, publicly available datasets. Most existing datasets are limited in scope, often containing only a small number of strength parameters and a small set of mix design variables. Many published datasets are region-specific and focus primarily on a limited range of mix designs, restricting their applicability to broader contexts. To overcome these limitations, a comprehensive and well-structured global dataset has been compiled, comprising 2188 UHPC mix designs from 168 research articles, useful for development of ML models and optimization of UHPC mixes for diverse structural and environmental applications.

## 3 Data Description

This dataset provides a detailed description of UHPC mixes incorporating SCMs, nano particles and sustainable fillers. The dataset is designed to comprehensively capture the composition, material types, and key physical properties relevant to UHPC and performance evaluation. The dataset is organized in a Microsoft Excel file names “UHPC Dataset.xlsx” which contains several worksheets. The dataset is organized in a tabular format in the “UHPC Dataset” worksheet, with each row representing a unique UHPC mix design. Columns include binder types (cement type, amount, grade), SCMs (e.g., silica fume, fly ash, GGBFS, metakaolin, glass powder, quartz powder, rice husk ash), nano particles (e.g., nano-silica, nano-calcium carbonate, nano-alumina, nano-titania), filler types (e.g., silica flour, steel slag, recycled brick powder), Fiber properties (type, amount, geometry, mechanical properties), aggregates (sand type, amount, maximum size), admixtures (superplasticizer), curing conditions (method, temperature, humidity and pressure), and measured workability, mechanical and durability properties. While the other worksheets the file contains the chemical compositions of Cement, SCMs, fillers present in the dataset. All material quantities are standardized (e.g., kg/m³ for mass, mm for size, MPa for strength, GPa for modulus) to ensure consistency and comparability across entries. Table 1 outlines the structure of the UHPC Dataset, listing each column name and corresponding unit.

**Table 1:** The UHPC dataset structure.

No	Column	Category	Name	Unit	No	Column	Category	Name	Unit	
1	A	Binder	Mix-ID		47	AU	Compressive Strength	Specimen	mm	
2	B		Cement Amount	kg/m^3^	48	AV		Testing Standard	–	
3	C		Cement Type	–	49	AW		1-day	MPa	
4	D		Cement Grade	MPa	50	AX		3-Day	MPa	
5	E	Supplementary Cementitious Materials (SCMs)	Silica Fume	kg/m^3^	51	AY		7-Day	MPa	
6	F		Fly Ash Amount	kg/m^3^	52	AZ		14-Day	MPa	
7	G		Fly Ash Type	–	53	BA		21 days	MPa	
8	H		Limestone Powder	kg/m^3^	54	BB		28-Day	MPa	
9	I		Quartz powder	kg/m^3^	55	BC		56-Day	MPa	
10	J		Glass powder	kg/m^3^	56	BD		90-Day	MPa	
11	K		Rice husk ash	kg/m^3^	57	BE	Elastic Modulus	Testing Standard	–	
12	L		Metakaolin	kg/m^3^	58	BF		Elastic Modulus	GPa	
13	M		GGBFS	kg/m^3^	59	BG	Tensile Strength Parameters	Testing Standards	–	
14	N		Slag Amount	kg/m^3^	60	BH		Split Tensile Strength	MPa	
15	O		Type of Slag	–	61	BI		First-cracking strength	MPa	
16	P	Nano Particles	Nano-CaCO_3_	kg/m^3^	62	BJ		Direct Tensile Strength	MPa	
17	Q		Nano-Al_2_O_3_	kg/m^3^	63	BK		Peak tensile strain	%	
18	R		Nano-TiO_2_	kg/m^3^	64	BL		Tensile Elastic Modulus	GPa	
19	S		Nano SiO_2_	kg/m^3^	65	BM		Ultimate Strain capacity	%	
20	T	Fillers	Filler	kg/m^3^	66	BN	Flexural Strength Parameters	Specimens	mm	
21	U		Type of Filler	–	67	BO		Testing Standard	–	
22	V	Sand	Sand Amount	kg/m^3^	68	BP		LOP	MPa	
23	W		Sand Type	–	69	BQ		MOR	MPa	
24	X		Sand Maximum Size	mm	70	BR		Peak Strength	MPa	
25	Y	Fibers	Type of Fiber	–	71	BS		Residual Strength (f600)	MPa	
26	Z		Amount of Fiber	kg/m^3^	72	BT		Residual Strength (f150)	MPa	
27	AA		Length of Fiber	mm	73	BU		Toughness	J	
28	AB		Diameter of Fiber	mm	74	BV	Durability Properties	Air Content/Air Void/ Porosity	Testing Standards	–
29	AC		Tensile Strength	MPa	75	BW			Air Content	%
30	AD		Nominal Young’s Modulus	GPa	76	BX			Air Void	%
31	AE	Synergetic Fibers	Type of Fiber	–	77	BY			Porosity	%
32	AF		Amount of Fiber	kg/m^3^	78	BZ		Water absorption	Testing Standards	–
33	AG		Length of Fiber	mm	79	CA			Water absorption	%
34	AH		Diameter of Fiber	mm	80	CB		Shrinkage	Testing Standards	–
35	AI		Tensile Strength	MPa	81	CC			28-day Shrinkage	µϵ
36	AJ		Nominal Young’s Modulus	GPa	82	CD			56-day Shrinkage	µϵ
37	AK	Water	Amount	kg/m^3^	83	CE		Freezing and Thawing	Testing Standards	–
38	AL	Superplasticizer	Type of Superplasticizer	–	84	CF			Freezing and Thawing	Cycles
39	AM		Amount	kg/m^3^	85	CG		Rapid Chloride Permeability	Testing Standards	–
40	AN	Curing Regime	Curing Method	–	86	CH			Rapid Chloride Permeability *	C
41	AO		Temperature	°C	87	CI		Surface Resistivity	Testing Standards	–
42	AP		Humidity	%	88	CJ			Surface Resistivity	(kΩ-mm)
43	AQ		Pressure	MPa	89	CK	Research Paper	Country of Origin		
44	AR	Workability	Testing Standard	–	90	CL		Reference details/Data sources		
45	AS		Slump	mm	91	CM		Year of Publish		
46	AT		Slump Flow	mm	92	CN		Research paper reference in dataset		

⁎ Rapid Chloride Permeability Test is measured in Coulombs (C).

### 3.1 Mix constituents

#### 3.1.1 Cement and its composition

**Fig. 1:**
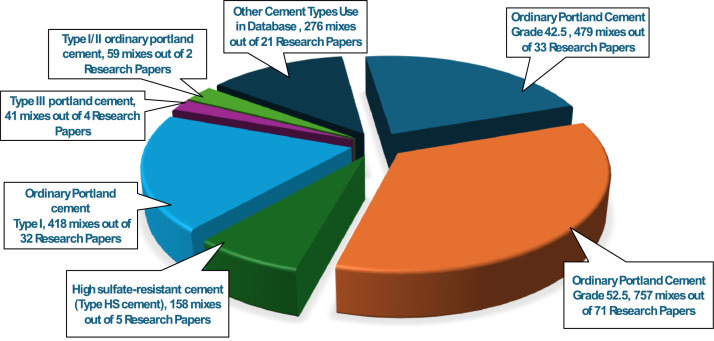
Type of Cement use in UHPC dataset.

The comprehensive UHPC dataset comprises 2188 mix designs, with every mix design utilizing cement as the primary binder. A wide variety of cement types are represented in Fig. 1, reflecting both regional practices and specific performance requirements in UHPC research. The most used cement is Ordinary Portland Cement (OPC) Grade 52.5, used in 757 mixes across 71 publications, followed by OPC Grade 42.5, which appears in 479 mixes from 33 publications. Other notable cement types include high sulfate-resistant cement (Type HS), OPC Type I, and Type III Portland cement, each contributing to a significant portion of the dataset. Special cement types, such as Type I/II, Portland cement Grade 53, blast furnace slag cement, GGBFS cement, low-alkali Portland cement, and white cement, are also included, though with lower frequency. The chemical compositions and ranges for all cement types are presented in Appendix – A, providing comprehensive information on oxide compositions and physical properties relevant to UHPC performance. A statistical summary of cement content is presented in Table 2.

**Table 2:** Statistical summary of cement content in the dataset.

Mean (kg/m^3^)	Standard Deviation (kg/m^3^)	Kurtosis	Skewness	Minimum (kg/m^3^)	Maximum (kg/m^3^)
789.13	199.52	2.67	0.405	170	1856.7

#### 3.1.2 Secondary cementitious materials and its composition

**Fig. 2:**
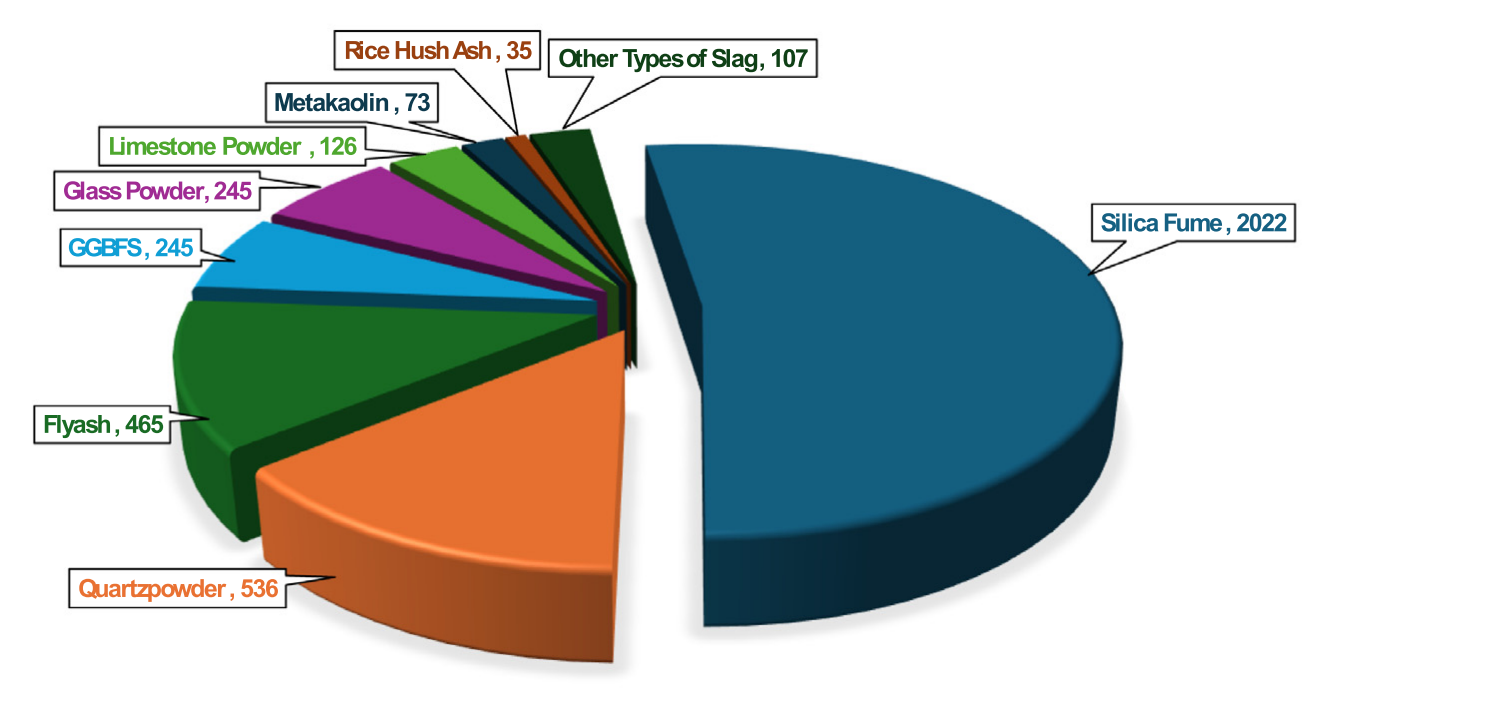
Different SCMS Types in the dataset.

SCMs are commonly used to the development of UHPC, serving as both sustainable and performance enhancing replacements for OPC [18]. In this dataset SCMs (Fig. 2) including fly ash, silica fume, GGBFS, metakaolin, rice husk ash, limestone powder, glass powder, and quartz powder are extensively utilized as partial replacements for OPC. These materials, often derived from industrial byproducts or processed minerals, not only contribute to resource efficiency, carbon footprint reduction and waste reduction but also impart significant improvements to the mechanical properties and durability performance of the UHPC. Notably, silica fume is the most frequently used SCM, present in 2022 mix designs, followed by quartz powder (536), fly ash (465), glass powder (245), GGBFS (245), limestone powder (126), metakaolin (73), other types of slag (107), and rice husk ash (35). Among the fly ash entries, 319 mixes utilize Class F fly ash and 134 employ Class C, reflecting the importance of distinguishing between these subtypes due to their differing chemical and pozzolanic characteristics. The incorporation of various slags such as steel slag, copper slag, phosphorous slag, and ultrafine slag powder further highlights the diversity of SCMs in the dataset. The widespread adoption of SCMs is driven by their ability to reduce the carbon footprint of concrete, enhance durability, improve workability, and utilize industrial byproducts, thereby enhancing sustainability and performance of UHPC. A statistical summary of SCMs used the dataset is tabulated in Table 3. Their chemical compositions and ranges are presented in Appendix – B.

**Table 3:** Statistical summary of SCMs used in the dataset.

	Mean (kg/m3)	Standard Deviation (kg/m3)	Kurtosis	Skewness	Minimum (kg/m3)	Maximum (kg/m3)	No. of mix Design	No. of research Paper
Silica Fume	195.39	75.77	5.99	1.03	18	617.65	2022	151
Flyash	275.11	185.76	0.83	1.06	26	1152	465	48
Limestone Powder	223.89	134.44	12.68	2.55	11	1058.2	126	26
Quartz powder	233.93	96.75	0.097	0.35	24.65	528	536	40
Glass Powder	331.67	208.19	1.99	1.45	20	1067	245	20
Rice husk Ash	172.52	88.45	4.57	1.85	46	481.06	35	8
Metakaolin	139.18	100.13	4.02	1.75	18	510	73	10
GGBFS	372.67	233.92	−1.21	0.46	21	768	245	40
Copper Slag	467.5	246.20	−1.23	0	85	850	60	1
phosphorous Slag	225	118.59	−1.2	0	75	375	5	1
Steel Slag	212.16	271.4	7.8	2.84	11	1100	27	6
Ultrafine Slag	55.38	–	–	–	–	–	16	1

#### 3.1.3 Nano materials

**Fig. 3:**
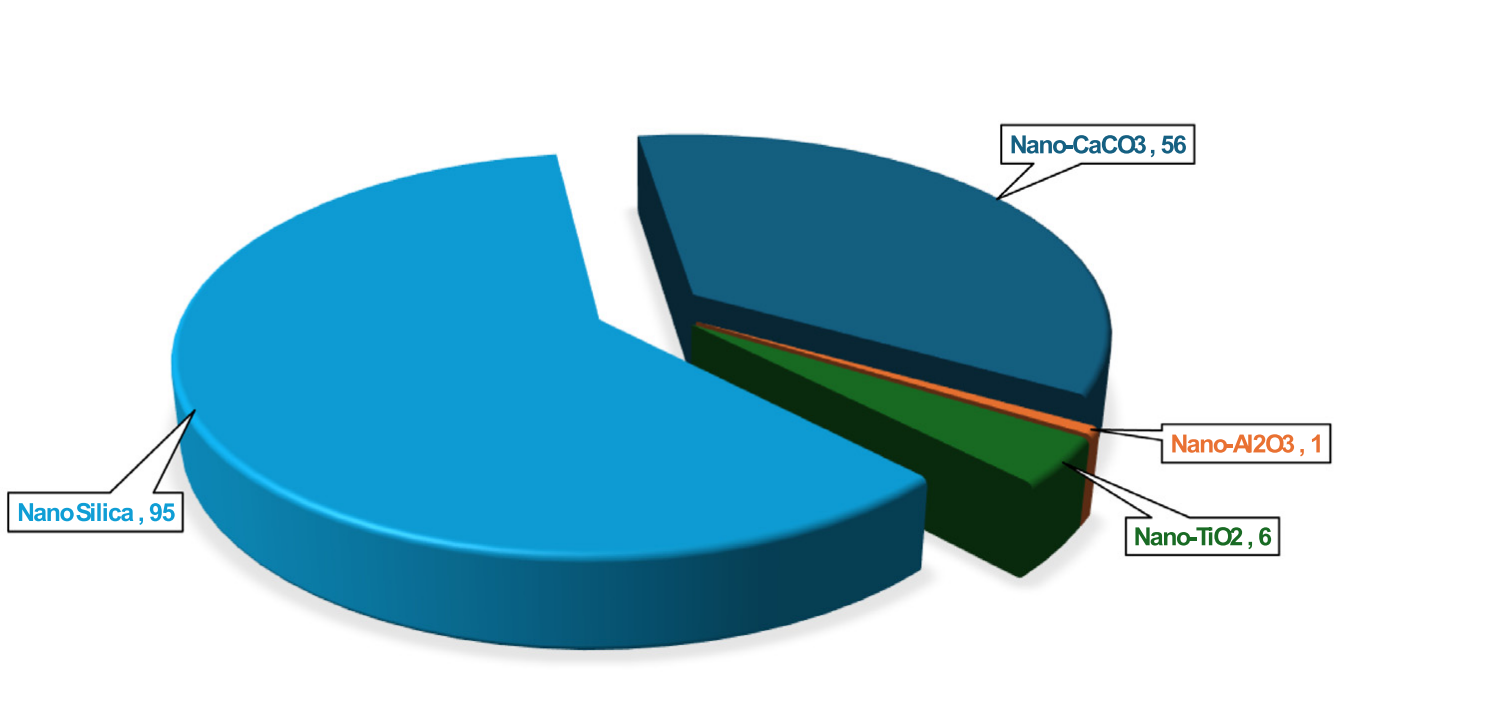
Different Types of Nano Particles in the dataset.

Nano particles are increasingly incorporated into UHPC to enhance its mechanical properties, durability, and microstructural characteristics [19]. In the compiled dataset, several types of nano particles are represented, including nano-calcium carbonate (Nano-CaCO₃), nano-alumina (Nano-Al₂O₃), nano-titania (Nano-TiO₂), and nano-silica (Nano-SiO₂). Each nano particle type is recorded with its dosage in the worksheet “UHPC Dataset”, allowing for detailed analysis of their effects across different mix designs. The statistical summary represented in Table 4 shows that nano-silica is the most frequently used, accounts for 95 mix designs, while nano-calcium carbonate appears in 56 mixes. Nano-alumina and nano-titania are less common, included in 1 and 6 mix designs, respectively. The dataset included all nano particle data with other mix design parameters, supporting comprehensive analysis and future optimization of UHPC formulations. Fig. 3 presents the distribution of different types of nano particles incorporated in the UHPC dataset.

**Table 4:** The detailed statistical analysis of Nano Particles used in the dataset.

	Mean (kg/m^3^)	Standard Deviation (kg/m^3^)	Kurtosis	Skewness	Minimum (kg/m^3^)	Maximum (kg/m^3^)	No. of mix Design	No of research Paper
Nano-CaCO_3_	23.99	12.71	2.97	1.3	6	69.1	56	6
Nano-Al_2_O_3_	63.1	–	–	–	63.1	63.1	1	1
Nano-TiO_2_	28.92	22.72	−1.04	0.63	4.8	63.1	6	2
Nano SiO_2_	19.47	12.18	1.52	1.14	4	63.1	95	9

#### 3.1.4 Sustainable fillers and sand

**Fig. 4:**
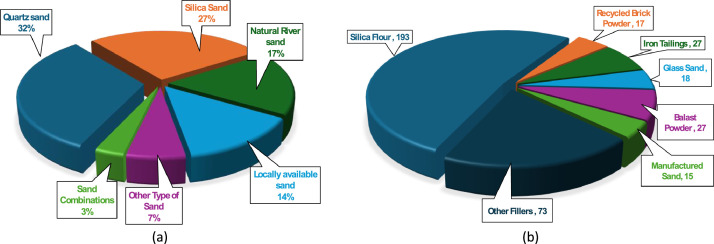
(a) Different Types of Sand and (b) Sustainable Fillers in the dataset.

Ultra-high performance concrete typically utilizes fine aggregates with particle sizes below 4.75 mm, as coarse aggregates are generally excluded to achieve a dense, homogeneous matrix and maximize mechanical performance. Fig. 4(a) shows that Quartz sand and silica sand are frequently used in UHPC dataset while others are less common. Furthermore, thirty research articles partially or completely replace sand in UHPC with more sustainable alternatives fillers. These include industrial byproducts and recycled materials such as iron tailings, recycled brick powder, blast furnace powder, and manufactured sand. Iron tailings, for example, are finely processed byproducts from iron ore extraction and can serve as a sand substitute. Recycled brick powder, derived from construction and demolition waste, is another sustainable filler that can replace sand, reduce landfill burden and conserve natural resources. These sustainable fillers, when properly graded and processed, can maintain or even enhance the mechanical and durability properties of UHPC. Appendix – C provides the chemical compositions and maximum size of all fillers presented in this study. Fig. 4(b) shows the distribution of different types of fillers used with Silica flour used in most (193) mixes design. Table 5 shows the statistical summary of sand and sustainable fillers used in the dataset.

**Table 5:** Statistical summary of sand and sustainable fillers in the dataset.

	Mean (kg/m^3^)	Standard Deviation (kg/m^3^)	Kurtosis	Skewness	Minimum (kg/m^3^)	Maximum (kg/m^3^)	No. of mix Design	No. of research Paper
Fillers	312.86	252.52	3.36	2	11	1255.8	370	30
Sand	931.26	247.03	1.76	−0.44	85	1994	2121	156

#### 3.1.5 Fibers

**Fig. 5:**
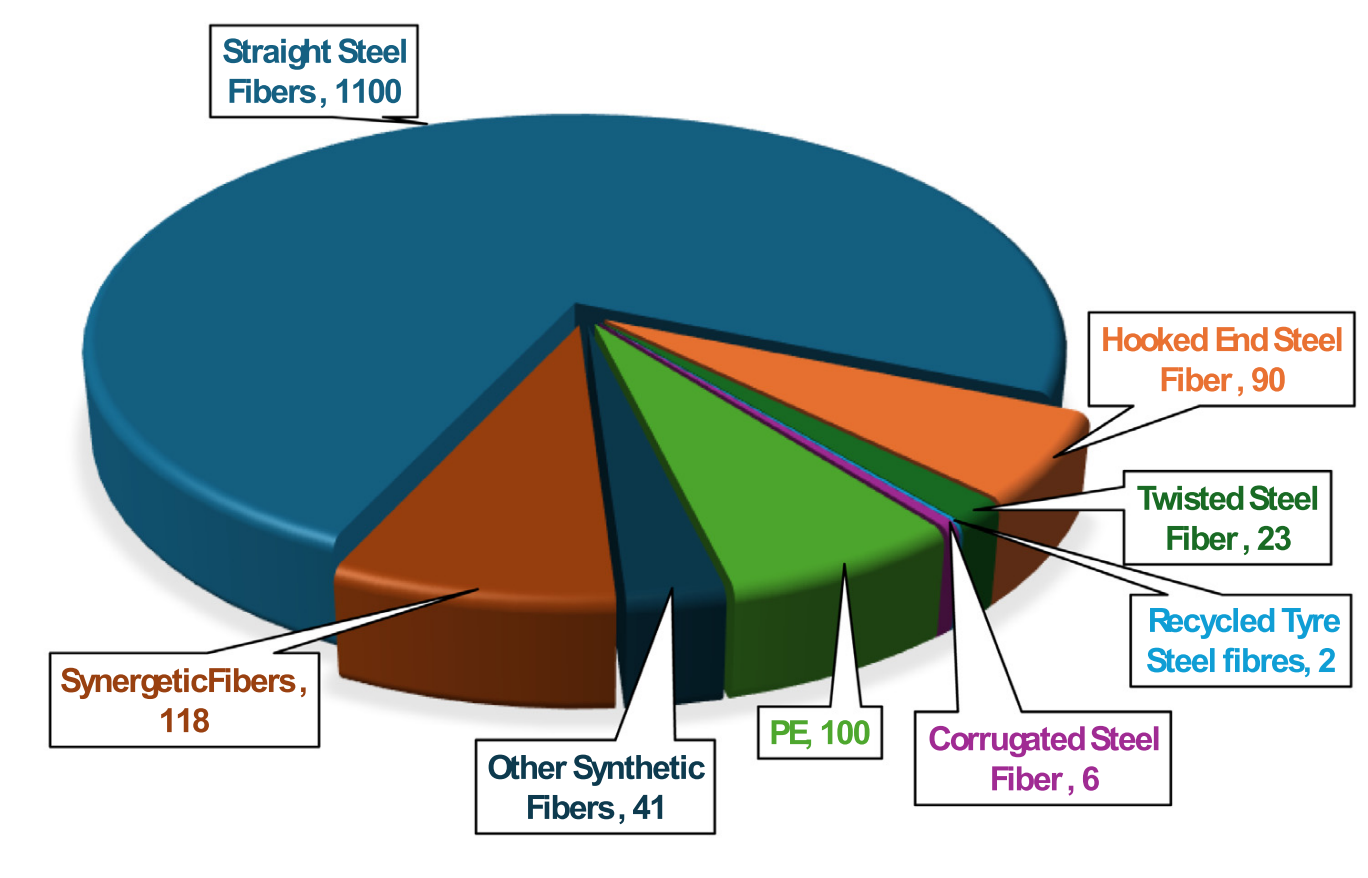
Different Types of fibers used in the dataset.

The UHPC dataset provides a comprehensive overview of the various fiber types incorporated into UHPC mix designs, highlighting their individual and synergistic effects on mechanical properties and durability performances. The dataset includes metallic fibers (including straight, hooked end, corrugated, and twisted steel fibers), synthetic fibers (including polyvinyl alcohol (PVA), polypropylene (PP), and polyethylene (PE)), mineral fibers (wollastonite), and carbon fibers shown in Fig. 5. The synergistic effect of hybrid fiber where combinations of different fiber types or geometries are used together is also documented, demonstrating improvements in tensile strength, ductility, and crack resistance compared to single-fiber systems. Table 6 provides the physical properties of each fiber type, including length, diameter, tensile strength and Young’s modulus. Table 7 presents a statistical summary of fiber usage included in the dataset.

**Table 6:** The physical properties of Fibers.

	Length (mm)	Diameter (mm)	Tensile Strength (MPa)	Young Modulus GPa
Micro Mineral Wollastonita Fibers	0.05	0.00333	–	–
Cellulose Fibre	2.1	0.018	900	–
Carbon Fiber	1	0.007	4300	250
Recycled Tyre Steel fibres	15	0.18	2000	–
Corrugated Steel Fiber	13	0.2	2800	–
Hooked End Steel Fiber	8 - 62	0.2 - 0.775	1200 - 2900	200 - 203
Straight Steel Fibers	6 - 60	0.032 - 0.55	1100 - 4200	200 - 210
Twisted Steel Fiber	8 - 30	0.3	2100 - 2428	200.00
Micro glass Fiber	13	0.018	2000	–
Polyethylene Fiber (PE)	12 - 36	0.015 - 0.03	2400 - 3900	42.80–145
Polypropylene Fiber (PP)	10	0.003	400	–
Polyvinyl Alcohol Fiber (PVA)	12 - 18	0.039 - 0.2	1000	29

**Table 7:** Statistical summary of fibre dosage used in the dataset.

	Mean	Standard Deviation	Kurtosis	Skewness	Minimum	Maximum
Fibers	141.15	76.93	9.76	1.76	6.50	780.00

#### 3.1.6 Water and superplasticizer

**Fig. 6:**
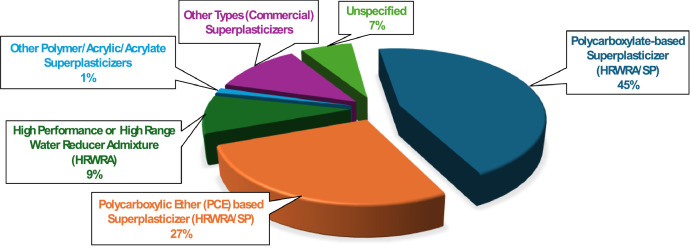
Different Types of Superplasticizers used in the dataset.

Superplasticizer is used in all mix designs in the dataset to achieve the required workability, especially given the low water-to-binder ratios of typical UHPC. The dataset records the dosage of superplasticizer for each mix, allowing for detailed analysis of its influence on fresh and hardened properties. The dataset comprises various types of superplasticizers, as shown in Fig. 6. Polycarboxylate-based superplasticizers (45 %) and PCE-based (27 %) dominate the dataset, followed by HRWRA (9 %), other commercial superplasticizers (11 %), unspecified types (7 %), and acrylic/acrylate-based superplasticizers (1 %). Table 8 provides a statistical summary of water and superplasticizer dosages.

**Table 8:** Statistical summary of water and superplasticizer used in the dataset.

	Mean (kg/m^3^)	Standard Deviation (kg/m^3^)	Kurtosis	Skewness	Minimum (kg/m^3^)	Maximum (kg/m^3^)
Water	194.59	42.81	1.97	1.38	110.00	355.15
Superplasticizer	31.75	17.70	4.75	1.39	2.64	151.70

### 3.2 Curing regimes

**Fig. 7:**
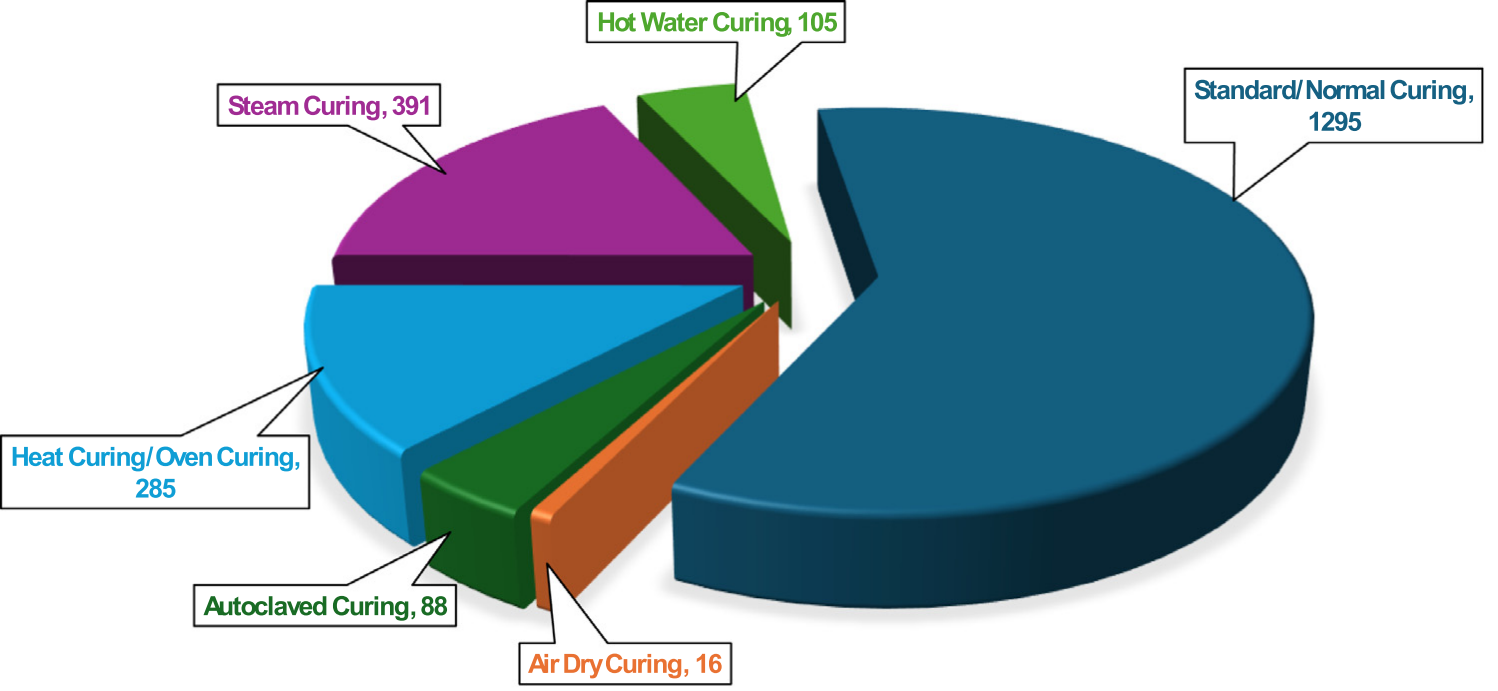
Different types of curing method.

Curing regime is an important factor in the development of UHPC, directly influencing microstructural evolution and the resulting mechanical and durability properties. In the compiled UHPC dataset, detailed records of curing temperature, humidity and, in the case of autoclave curing pressure values and duration are provided for each mix, enabling a comprehensive analysis of the curing conditions applied. The selection and optimization of curing regimes are tailored to the specific performance requirements of UHPC, with high-temperature and high-humidity (such as autoclaved, steam, and heat curing) commonly used to maximize early-age strength and durability. Fig. 7 illustrates the different types of curing method used . While Table 9 presents the range of temperature, humidity and pressure associated with each curing method.

**Table 9:** The mean and range of temperature, humidity and pressure of each curing method.

Curing Regime	Temperature ( °C)		Humidity ( %)		Pressure (MPa) and Duration (hrs)
	Mean	Range	Mean	Range	Range
Standard/Normal Curing	20.99	20 - 30	90.29	50 - 100	–
Air Dry Curing	24	20 - 26	56.25	55 - 60	–
Autoclaved Curing	193.3	85 - 210	–	–	1.2 MPa for 4 hrs - 2 MPa for 8 hrs
Heat Curing /Oven Curing	98.77	70 - 200	97.48	85 - 100	–
Steam Curing	88.61	65 - 150	97.88	95 - 100	–
Hot Water Curing	86.95	65 - 90	–	–	–

### 3.3 Workability, mechanical properities and durability performances

The global UHPC dataset provides a comprehensive overview of the workability, mechanical properties, and durability performance of UHPC mixes in the dataset. These properties are critical for evaluating the fresh behavior, strength development, and long-term performance of UHPC across diverse mix designs and curing regimes. Table 10 shows the statistical summary of Rheology, mechanical properties and durability performances. Workability data, including slump and slump flow, provide insights into the fresh workability of UHPC across a diverse set of mixes. The mechanical properties of UHPC mixes in the dataset is measured by compressive strength at various ages with specimens, as well as detailed records of tensile strength parameters (including splitting and direct tensile strength, first cracking strength, peak tensile strain, strain capacity, tensile elastic modulus), flexural strength (modulus of rupture MOR, limit of proportionality LOP, Peak flexural strength and Toughness), and elastic modulus. Specimen dimensions of mechanical properties differ across the studies included in the dataset, and such variations can considerably affect the reported mechanical strengths. To maintain transparency, these specimen sizes are explicitly documented in the dataset, allowing users to account for this variability when conducting comparisons or predictive modeling. Appendix D specifically provides information about the specimens used for testing compressive and flexural strength. Also, the dataset explicitly records the testing standards associated with each reported property, including ASTM, EN, and equivalent national codes. Appendix E provides a comprehensive list of the testing standards applied for evaluating the mechanical, durability, and workability properties of UHPC.

**Table 10:** Statistical summary of Workability, mechanical properties and durability performances.

		Quantity	Mean	Standard Deviation	Kurtosis	Skewness	Minimum	Maximum
Workability	Slump (mm)	33	128.33	99.69	3.59	1.95	19	444
	Slump Flow (mm)	1161	212.40	55.51	0.11	0.20	52.6	390
Compressive Strength	1-day (MPa)	123	55.78	14.10	0.53	−0.33	4.1	82
	3-Day (MPa)	273	86.93	21.67	1.58	0.89	38	163
	7-Day (MPa)	754	106.94	27.80	0.14	0.40	39	201
	14-Day (MPa)	130	120.80	24.83	0.03	0.39	70.66	193
	21 days (MPa)	10	159.54	19.07	0.19	−1.22	126	176.4
	28-Day (MPa)	2073	150.25	36.56	0.85	0.76	80	338
	56-Day (MPa)	217	152.42	22.82	−0.23	−0.51	78	202
	90-Day (MPa)	341	154.81	26.14	−0.36	0.05	89	225
Elastic Modulus	28-Day (GPa)	243	43.86	8.23	2.90	-1.24	15	68
Tensile Strength Parameters	Split Tensile Strength (MPa)	237	15.74	6.76	0.01	0.60	3.8	39.8
	First Cracking Strength (MPa)	103	7.29	2.31	−0.48	0.48	3.06	13.3
	Direct Tensile Strength (MPa)	280	11.53	5.08	12.90	2.81	4.4	43.22
	Peak tensile strain ( %)	42	3.34	2.78	−1.21	0.36	0.088	8.2
	Tensile Elastic Modulus (GPa)	35	44.52	16.97	−1.46	−0.49	17.2	64.5
	Strain Capacity ( %)	130	3.04	2.94	−0.24	0.99	0.03	10.15
Tensile Strength Parameters	LOP (MPa)	66	15.51	3.51	6.58	−0.05	1	28.64
	MOR (MPa)	140	26.01	10.07	−0.81	0.29	6.1	50.9
	Peak Strength (MPa)	1024	25.91	9.49	0.01	0.18	3.1	64
	Residual Strength (f600) (MPa)	68	20.27	7.30	−0.79	0.48	5.9	35.33
	Residual Strength (f150) (MPa)	73	15.55	9.91	0.61	1.23	3.3	46.11
	Toughness (J)	202	94.58	133.77	4.38	2.32	1	605.6
Durability Property	Air Content %	47	3.332553	1.073982	0.042283	0.329794	1.4	6.3
	Air Void %	38	4.513158	1.253788	0.210081	0.507986	2.2	7.9
	Porosity ( %)	239	6.223389	5.167403	0.951409	1.205601	0.69	25.89
	Water absorption ( %)	23	1.325391	0.618443	−0.45665	0.683241	0.43	2.637
	28-day Shrinkage (µstrain)	112	464.3678	412.6693	4.628304	2.111068	6	2100
	56-day Shrinkage (µstrain)	66	358.5303	339.5936	20.52698	4.216599	25	2300
	Freezing and Thawing (Cycles)	22	76.81818	40.05684	−1.59466	−0.67932	17.7	109
	Rapid Chloride Permeability Test (Coulombs)	108	410.3278	559.5988	2.081416	1.887589	9	1970
	Surface Resistivity (kΩ-mm)	13	460.6154	67.77971	0.735942	1.280231	386	603

**Fig. 8:**
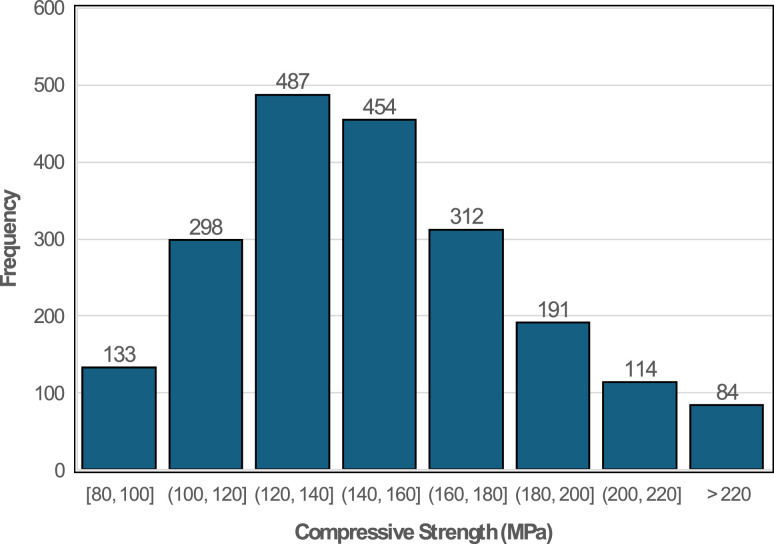
Distribution plots of compressive strength.

The 28 days compressive strength values fall within the range of 80 and 330 MPa at 28 days. The distribution of compressive strength shown in Fig. 8 highlights that the greatest frequency is observed in the 120–160 MPa interval, highlighting that most UHPC mixtures achieve exceptionally high strength at 28 days. The distribution of results is positively skewed (skewness 0.76), consistent with the high strength expected in UHPC mixes. In terms of durability performances, the dataset captures a comprehensive set of indicators, including porosity, air content, shrinkage, freeze-thaw resistance, rapid chloride permeability, surface resistivity, and water absorption. Most UHPC mixes show low porosity and strong resistance to chloride penetration due to dense microstructure facilitated by SCMs and nano additives. The availability of shrinkage data across different curing regimes allows for the evaluation of crack and dimensional stability over time. Additionally, results for freeze-thaw and abrasion resistance highlight the suitability of UHPC for demanding harsh environmental exposures and applications subject to heavy wear. This comprehensive suite of data supports both comparative analysis and development of ML model for the optimization of UHPC design that enhanced long-term durability performances.

### 3.4 Global and year wise distribution of UHPC publications

This section presents an overview of the country-wise and year-wise distribution of publications included in the UHPC dataset. Table 11, Table 12 summarize the distribution of publications contributing to the UHPC mix design dataset by country and year-wise, respectively. A total of 27 countries are represented, reflecting the international scope and diversity of the dataset. The largest contributions come from China and the USA, which account for 36.79 % and 15.63 % of the total mix designs, respectively. Beyond these major contributors, the dataset includes substantial inputs from countries such as Turkey, Korea, India, Malaysia, Canada, Saudi Arabia, Netherlands, Iran, France, Singapore, and Germany. Notably, the dataset incorporates the most recent publications ranging from 2006 to 2025. This wide range representation in the dataset means it covers many different raw materials, environmental conditions, and construction practices used across different regions. Having such a globally diverse dataset strengthens the reliability and applicability of data analysis, particularly for research comparing regional trends or for developing ML models to predict UHPC properties.

**Table 11:** Regional contribution of publications.

Country	Publication count	Mix design count	Mix design count proportion ( %)
Algeria	1	2	0.09
Brazil	1	1	0.05
Austria	1	4	0.18
Lithuania	1	4	0.18
Switzerland	1	4	0.18
Croatia	1	8	0.37
Pakistan	1	10	0.46
Vietnam	2	10	0.46
UK	2	14	0.64
Italy	2	14	0.64
Australia	3	16	0.73
Hong Kong	1	16	0.73
Cyprus	2	17	0.78
Germany	2	20	0.91
Singapore	5	26	1.19
France	2	28	1.28
Iran	3	41	1.87
Netherlands	5	53	2.42
Saudi Arabia	5	73	3.34
Canada	5	79	3.61
Malaysia	5	79	3.61
India	4	92	4.20
Portugal	4	136	6.22
Korea	11	140	6.40
Turkey	7	154	7.04
USA	19	342	15.63
China	72	805	36.79

**Table 12:** Year-wise contribution of publications.

Year	Publication count
2006	2
2007	1
2008	5
2010	6
2011	6
2012	6
2013	5
2014	9
2015	12
2016	16
2017	17
2018	23
2019	18
2020	8
2021	12
2022	6
2023	5
2024	9
2025	2

## 4 Experimental Design, Materials and Methods

The dataset presented in this study was compiled to capture the mix design, material constituents, and performance characteristics of UHPC containing SCMs, nano additives, and sustainable fillers. Data acquisition involved a multi-stage process to ensure accuracy, consistency, and comprehensive coverage of relevant literature.

(1) **Literature collection:** First, an extensive literature review was conducted using academic databases such as Scopus, Web of Science, and ScienceDirect. Keywords including "ultra-high performance concrete," "supplementary cementitious materials," "fly ash," "nano particles” and "mechanical properties" were used to identify relevant publications. Only peer-reviewed journal articles published between 2006 and 2025 were considered. Publications were carefully selected for creating the dataset to ensure that for all mix designs included, adequate details of the UHPC mix proportions, use of one or more SCMs or nano additives, mechanical properties and durability performances are available.
(2) **Data Extraction:** Data was extracted directly from text and tables in the selected publications. For graphical data, the free open-source software is employed to extract and validate data. All values were double checked for accuracy. Each UHPC mix design was recorded as a single row in the dataset with a unique ID referencing its source.
(3) **Data Organization:** The raw data were compiled in a Microsoft Excel file with many worksheets using a structured format. Each row in the "UHPC Dataset" worksheet represents one unique UHPC mix, while columns represent different variables grouped under categories such as: Cement (type, amount, grade), SCMs (e.g., silica fume, fly ash, GGBFS, metakaolin),Nano materials (e.g., nano-silica, nano-CaCO₃, nano-Al₂O₃, nano-TiO₂), Fillers and aggregates (type, size, amount),Fiber properties (type, geometry, content, mechanical properties), Water and superplasticizer dosage, Curing (method, temperature, humidity and pressure (Autoclave Curing)), Workability (slump, slump flow), Mechanical properties (compressive strength at 1, 3, 7, 14, 21, 28, 56, and 90 days, elastic modulus, flexural and tensile strengths), Durability performances (porosity, shrinkage, RCPT, water absorption, etc.). Each record in the dataset includes the country of origin, publication year, and research article ID. Mixes without sufficient property or mix data were excluded.
(4) **Data Standardization:** All material quantities were standardized to consistent metric units (e.g., kg/m³ for mass, mm for size, MPa for strength, GPa for modulus). Where SCMs or nano particles were reported as a percentage of binder or cement, values were converted to absolute mass per unit volume using:
  $$\text{Component}\;\left(\text{kg}/m3\right)=\frac{\text{Component}\;\left(\%\right)\;\times \;\text{Total}\;\text{binder}\;\left(\text{kg}/m3\right)}{100}$$
(5) **Category Unification:** Similar materials (e.g., different types of steel fibers, natural/recycled aggregates) were grouped under unified categories for streamlined analysis.
(6) **Composition Details:** Supplementary worksheets that contain chemical composition data for cements, SCMs, and fillers, reported in terms of major oxides (e.g., CaO, SiO₂, Al₂O₃, Fe₂O₃, in terms of percentage) from the corresponding publications are also included.

## Limitations

The dataset only covers UHPC mixes with compressive strengths ranging from 80 to 300 MPa at 28 days curing age. Mix designs with strengths outside this range are not represented, which may restrict the applicability outside the collected data range. Second, despite efforts to extract complete data, some studies lacked consistent reporting of properties such as tensile strain capacity, durability metrics, or specific curing conditions, leading to partial records for some entries.

## Ethics Statement

The authors confirm that they have read and followed the ethical requirements for publication in Data in Brief. The current work does not contain any studies with human participants, animals performed, or any data collected from social media platforms by any of the authors.

## Credit Author Statement

**Umair Jalil Malik:** Conceptualization, Methodology, Data curation, Writing - original draft preparation. **Damith Mohotti:** Conceptualization, Supervision, Investigation, Writing - reviewing and editing. **Huadong Mo:** Conceptualization, Supervision, Investigation, Writing - reviewing and editing. **Chi King Lee:** Conceptualization, Supervision, Investigation, Writing - reviewing and editing.

## Figures and Tables

**Table:** **Appendix A:** Composition of different types of cement

	Research Paper		CaO	SiO₂	Al₂O₃	Fe₂O₃	K₂O	Na₂O	SO₃	MgO	MnO	TiO₂	Mn₃O₄	P₂O₅	Cl⁻	LOI	Specific gravity
OPC Grade 42.5	29	Mean	61.68	21.11	5.58	3.32	0.73	0.36	2.60	2.16	0.10	0.20			0.01	2.90	3.13
		Range	53.32 - 66.8	15.4 - 29.67	3.74 - 8	1.8 - 5.4	0.13 - 1.2	0.07 - 0.73	0.58 - 3.66	0.86 - 4.1	0.1 −0.1	0.09 −0.3			0.001 −0.013	1 - 11.6	3.1 - 3.15
OPC Grade 52.5	58	Mean	63.45	20.77	4.74	3.07	0.64	0.30	2.81	1.76	0.10	0.24	0.08	0.22	0.05	2.72	3.14
		Range	54.6 - 69.02	17.33 - 29.67	0.21 - 8.33	0.36 - 5.23	0.04 - 1.06	0.08 - 1	1.74 - 4.1	0.6 - 5.31	0.01 0.22	0.07 −0.6	0.02 - 0.1	0 - 0.74	0.005 - 0.11	0 - 11.6	3.1 - 3.25
OPC Type I	27	Mean	63.47	20.65	5.25	3.43	0.60	0.20	2.56	2.30	0.10	0.29		0.25		1.76	3.15
		Range	61.33 - 76.2	11.8 - 22.09	1.88 - 6.4	2.9 - 4.34	0.35 - 1.51	0.14 - 0.3	1.86 - 3.64	0.82 - 3.3	0.06 - 0.12	0.192		0.06 - 0.6		0.7 - 2.5	3.15 - 3.18
High Sulfate-resistant Cement	5		65.6	22	3.5	4.3	0.8	0.07	2.3	1.9		0.2				1	3.21
Type III Portland cement	3		64.5	19.72	5.1	2.76		0.33	3.25	2.3					2.6		3.15
Type I/II ordinary Portland cement	2		63.8 - 64.3	20.2 - 22.3	3.4 - 4.3	2.8 - 3.1	0.47	0.22	0.35 - 2.5	1.6 - 2.3						0.88	3.15
Blast furnace cement III/40	1		52.14	24.94	7.5	2.62	0.51	0.09	2.57	5.34						2.63	
Type I/II low-alkali Portland cement based on	1		63.9	20.3	4.6	3.4	0.38	0.23	2.86	1.91						2.24	
Portland cement (PC) with 28 days of compressive strength of 68.9 MPa	1		65.1	20.6	5	4.4			2.2	0.6						1.3	

**Table:** **Appendix B:** Composition of different types of SCMs.

		CaO	SiO₂	Al₂O₃	Fe₂O₃	K₂O	Na₂O	SO₃	MgO	MnO	TiO₂	Mn₃O₄	P₂O₅	Cl⁻	LOI	Specific Gravity
Silica Fume	Mean	0.64	94.54	0.53	0.69	0.85	0.33	0.37	0.61	0.11	0.06	0.38	0.18	0.90	1.80	2.23
	Range	0.0001 - 2.25	82.22 - 99.8	0 - 2.22	0.02 - 2.44	0.01 - 4.12	0 - 1.3	0 - 0.99	0 - 3.41	0 - 0.654	0 - 0.3	0.01 - 1.04	0 - 1.31	0.0011 - 2.6	0 - 6	2.1 - 2.6
Fly Ash Class C	Mean	24.48	38.31	20.80	6.88	1.07	2.23	1.29	2.79	0.25	1.45		1.15	0.55	1.70	2.67
	Range	16.05 - 28.1	35.8 - 42.1	14 - 24.8	4.6 - 22.9	0.57 - 2.09	0.4 - 9.2	0 - 3.3	1.8 - 4.3	0.25 - 0.25	1.45 - 1.45		1.15 - 1.15	0.3 - 1.3	1.3 - 2.1	2.58 - 2.7
Fly Ash Class F	Mean	6.38	51.21	28.39	6.51	1.45	0.91	1.18	1.41	0.05	1.04	0.47	0.34	0.02	2.23	2.48
	Range	1.8025 - 14.45	42.3 - 62.08	18.56 −38.01	2.784 - 12.49	0.5 - 2.26	0.33 - 2.23	0.2 - 4.24	0.23 - 3.79	0.019 - 0.104	0.312 - 1.67	0.11 - 1.2	0 - 0.89	0.0149 - 0.02	0.168 - 7.74	2.24 −2.9
Limestone	Mean	66.23	2.11	0.67	0.68	0.97	0.18	0.11	1.20		0.06	1.25	0.16	0.12	36.67	2.67
	Range	49.27 - 89.56	0.07 - 4.36	0 - 1.06	0.02 - 1.6	0 - 7.708	0.01 - 0.627	0.02 - 0.48	0.13 - 2.891		0.06 - 0.06	0 - 1.605	0 - 0.241	0 - 0.241	0.01 - 46.62	2.64 - 2.7
Quartz powder	Mean	0.57	98.41	0.29	0.13	1.44	0.15	0.45	0.16	0.00	0.10	0.25	0.54	0.13	0.74	2.69
	Range	0.01 - 1.61	95.5 - 99.8	0.05 - 0.75	0.01 - 0.74	0.02 - 3.5	0.02 - 0.25	0 - 0.9	0 - 0.21	0 - 0.01	0 - 0.25	0.25 - 0.25	0.003 - 1.51	0.13 - 0.13	0.02 - 3.45	2.65 - 2.73
Glass Powder	Mean	9.69	75.24	1.92	0.70	1.38	12.93	0.14	1.62	0.05	0.05		0.01	0.46	2.70	
	Range	4.1 - 11.3	55.6 - 99	0.24 - 3.6	0.046 - 1.77	0.28 - 8.1	10.46 - 14.91	0.03 - 0.25	0.51 - 3.45	0.02 - 0.1	0.04 - 0.06		0 - 0.02	0.05 - 0.6	0.09 - 13.55	
Rice Hush Ash	Mean	1.00	89.11	0.34	0.34	3.50	0.09	0.42	0.61	0.49			0.75		3.19	2.19
	Range	0.71 - 1.35	87.4 - 92	0.19 - 0.61	0.17 - 0.63	1.87 - 4.6	0 - 0.2	0.39 - 0.47	0.47 - 0.8	0.28 - 0.7			0.75 - 0.75		0.76 - 4.6	2.19 - 2.19
Metakaolin	Mean	0.49	54.12	40.64	1.24	0.62	0.39	0.10	0.59		1.50		0.61		1.13	2.55
	Range	0.03 - 1.15	46.65 - 58.1	35.14 - 45.77	0.51 - 3.87	0.1 - 1.42	0.02 - 1.52	0.03 - 0.15	0.07 - 2.66		1.5 - 1.5		0.61 - 0.61		0.59 - 1.85	2.5 - 2.6
GGBFS	Mean	37.33	36.07	12.14	2.20	0.95	0.27	1.44	7.71	0.40	0.67	0.02	0.06	2.76	1.30	2.89
	Range	32.13 - 45.86	25.52 - 40.9	0.72 - 14.89	0.2 - 18.9	0.1 - 1.44	0.2 - 0.46	0.06 - 7.4	3.41 - 10.8	0.3 - 0.5	0.5 - 0.8	0.01 - 0.03	0.01 - 0.11	0.04 - 5.1	0.1 - 3.52	2.78 - 2.91
Steel Slag	Mean	44.14	15.80	4.13	20.73	0.06	0.26	0.34	5.20		3.53	1.04		1.63	8.59	3.15
	Range	41.001 - 48.66	12.67 - 19.11	0.84 - 7.46	18.01 - 22.69	0.06 - 0.06	0.119 - 0.41	0.344 - 0.344	2.8 - 6.138		2.66 - 4.393	1.042 - 1.042		1.5 - 1.76	8.59 - 8.59	2.81 - 3.75
Phosphorous Slag		46.73	42.77	3.34	0.51	0.64	0.25	0.60	2.27			0.14		1.81		
Copper Slag		2.00	27.00	3.00	57.00	0.19	0.20		0.29							
Ultrafine Slag Powder		41.80	33.10	13.70	0.70	0.50	0.20	0.70	4.90							

**Table:** **Appendix C:** Composition of different types of sustainable fillers.

	CaO	SiO₂	Al₂O₃	Fe₂O₃	K₂O	Na₂O	SO₃	MgO	MnO	TiO₂	Mn₃O₄	P₂O₅	Specific gravity	Max Size
Basalt Powder	14.84	48.71	14.58	4.9	1.83	3.45	1.62	2.76						
Cement kiln dust	49.3	17.1											2.79	
Glass Sand	11.3	73	0.4	1.5	0.5	13	–	1.2		0.04				
Gold mine tailing	3.534	60.426	14.045	6.663		0.627	0.226	2.498	0.219	1.109		0.407		0.6
Ground Granite Powder (GGP)	2.31	83.21	2.02	1.76	3.39	4.72		0.54						
Iron Tailings	12.74 - 21.67	32.76 - 52.06	17.14 - 6.896	9.13 - 16.92	0.3 - 2.03	0.97 - 1.513	9.486	3.68 - 6.052	0.45	0.25 - 1.013	0.176		2.94	0.6 - 1
Natural pozzolana (NP)	8.06	42.13	15.33	12.21	0.84	2.99		8.5		0.6		1.37	3	
Pumicite	0.87	63.86	31.11	1.06	0.09	1.08	0.05	0.18					2.6	
Recycled Fine Powder	20.86	46.84	15.49	6.95	2.76	1	2.05	2.66		0.77				
Steel slag	43.37	19.11	7.46	18.01		0.41		5.17	2.66			1.76		1.25
Silica Flour	0.3	98 - 98.8	0.2	0.1	–	–		0.2						
Dolomite														2
Recycled Brick Powder														0.15
Manufactured Sand														1.25
Rejected Flyash													2.19	1.18

**Table:** **Appendix D:** The specimens used for testing compressive and flexural strength.

Compressive Strength Specimens		Flexural Strength Specimens	
Compressive Specimens (mm^3^)	Quantity	Flexural Specimens (mm^3^)	Quantity
100×100×100	224	100×100×300	19
100×100×300	11	100×100×400	115
150×150×150	27	100×100×500	23
40×40×160	495	25×25×100	4
40×40×40	226	25×50×508	15
40×40×80	36	35×70×330	3
50×50×50	674	40×40×160	696
50×50×100	6	50×50×200	18
70×70×70	143	70×70×280	29
Cube (Not Specify)	2	70×70×350	9
Cylinder Ø 75×150 mm	95	75×75×300	4
Cylinder Ø 100×100	31	75×75×305	24
Cylinder Ø 100×200	99	75×100×400	16
Cylinder Ø 110×220	7	76 × 76 × 419	6
Cylinder Ø 150×300	12	76.2 × 76.2 × 304	30
Cylinder Ø 50×100	10	76×102×406	16
Cylinder Ø 50×50	4		

**Table:** **Appendix E:** Summary of testing standards used for UHPC properties.

List of Testing Standards	
Properties	Testing Standards
Slump	ASTM C143, Chinese standard GB/T 50,080–2016, GB/T50081–2002
Slump Flow	ASTM C 1437, AS 1012.3.5, ASTM C230, ASTM C1611, Chinese standard GB/T 2419–2005, Chinese standard GB/T 50,080–2016, GB/T50081–2002, 2002; ASTM C143–C143M-15a, 2015, BS EN 445: 2007, BS-EN–1015–3, BS EN 4551, Deliverable ARCHES D06 (2009), EFNARC, CECS (2009), EN–12,350–8 (2010), GB/T17671–2021, ASTM C1856
Compressive Strength	BS1881, ASTM C469, ASTM C 109, BS EN 196, BS EN 12,390, EN 1015, EN 12,390, ASTM C39, EN 1992, Chinese standard GB-T50081–2002, GB/T 17,671–1999, GB/T 31,387–2015, BS EN 1881, ASTM C 116, FHWA-HRT-06–103, AS1012.9, ASTM C 348, ASTM C1856, GB177–85, ASTM C1609, IS 4031–1988, Chinese standard test specification (CECS 2009), Swiss standard SIA 2052–2016
Elastic Modulus	ASTM C 469, ASTM C39, AS 1012.17,Chinese standard GB-T50081–2002, BS 1881–121, EN 12,390, GB/T17671–2021, EN 13,412:2007, DIN 1048–5:1991–06, DIN EN 12,390–3:2009–07 and DIN 1045–1
Split Tensile Strength	AS 1012.10, ASTM C496, EN 12,390–6, GB/T 31,387–2015, GB/T50081–2002, BS 1881: 1983, Leutbecher Thesis
Direct Tensile Strength	JSCE 2008
Flexural Strength	ASTM C348, ASTM C78, ASTM C 1018, ASTM C1609, ASTM C 116, ASTM E 399–12, ASTM C1856, ASTM C109, AS 1012.11, Chinese standard GB-T50081–2002, GB/T 17,671–1999 (similar to ISO 697:1989), GB177–85, NF EN196–1, EN 1015–11, BS1881, BS EN 12,390 Part5, Chinese standard test specification (CECS 2009), RILEM 50-FMC/198 Technical Committee, JCI-S–002–2003 standard, JSCE SF-4 for Flexural toughness
Air Content, Air Void and Porosity	ASTM C642, NT Build 492, ASTM C1202, ASTM C185, ASTM C457, ASTM C 231, EN 1015–7:2000, CEMBUREAU
Water Absorption	ASTM C-642, AS/NZS 4456.17, BS BS1881: Part 122, IS 3495 (Part 2) (BSI 1992)
Shrinkage	ASTM C157, ASTM C1698, JTG E30–2005, BS EN 12,617–4, DIN 52,450
Freezing and Thawing	ASTM C666, RILEM TC 176-IDC
Rapid Chloride Permeability	ASTM C1202
Surface Resistivity	AASHTO T 358

## Data Availability

Mendeley DataGlobal Dataset of UHPC Mix Designs with Supplementary Cementitious Materials, Nano Additives, and Sustainable Fillers (Original data)
